# Transesophageal echocardiography in robot-assisted mitral valve repair for Barlow’s disease: usefulness for predicting artificial ring size and artificial chordae length using the loop technique

**DOI:** 10.1186/s40981-020-00363-2

**Published:** 2020-07-25

**Authors:** Musashi Yahagi, Takuma Maeda, Hiroko Kanazawa, Kenji Yoshitani, Yoshihiko Ohnishi

**Affiliations:** 1 Department of Anesthesiology, National Cerebral and Cardiovascular Center, 6-1 Kisibeshinmachi, Suita, Osaka, 564-8565 Japan; 2 Division of Transfusion Medicine, National Cerebral and Cardiovascular Center, 6-1 Kisibeshinmachi, Suita, Osaka, 564-8565 Japan

**Keywords:** Transesophageal echocardiography, Barlow’s disease, Mitral valve repair, Loop technique, Robotic surgery

## Abstract

**Background:**

There is no fully recommended methodology for surgery for Barlow’s disease. Various methods have been proposed. The aim of this study was to investigate the effectiveness of transesophageal echocardiography (TEE) measurements for selecting the optimal annuloplasty ring size and determining the length of artificial chordae in patients with Barlow’s disease who underwent robot-assisted mitral valvuloplasty (R-MVP).

**Methods:**

Ten patients were included. Before R-MVP, the anesthesiologist used TEE to predict the optimal annuloplasty ring size and artificial chordae lengths that would reduce mitral regurgitation. The anesthesiolosist’s predict ring size was not presented to the surgeon intraoperatively.

**Results:**

In 70% (7/10) of cases, the surgeon performed mitral valve repair in full match with the anesthesiologist’s repair plan. Mitral regurgitation was controlled in 85% (6/7) of cases. In three cases, the predict annuloplasty ring size and artificial chordae length were not match between anesthesiologist and surgeon. After the operation, 90% (9/10) of patients had no residual mitral regurgitation.

**Conclusions:**

Anesthesiologist’s TEE measurements were useful for selecting the optimal annuloplasty ring size and artificial chordae length during R-MVP. TEE can play an important role in robot-assisted, minimally invasive cardiac surgery for mitral regurgitation with extensive and complex prolapse, such as in Barlow’s disease.

## Background

Minimally invasive cardiac surgery (MICS) has become widespread. Many institutions currently perform minimally invasive mitral valvuloplasty (MICS-MVP), with good results [1]. Recently, robot-assisted mitral valvuloplasty (R-MVP) has made it possible to perform plastic surgery for complex mitral valve prolapse; however, to choose MICS in cases of Barlow’s disease is challenging [2–5]. In Barlow’s disease, the repair process is complicated, and a cardiac arrest time is extended, so valve replacement is more likely to be selected than plastic surgery [6]. In the complex procedure of repairing the mitral valve in Barlow’s disease, precise sizing of the mitral annulus ring and artificial chordae is crucial. The loop technique by Mohr et al. and the concept of “respect rather than resect” by Perier et al. have been accepted in recent years [6–8]. The emphasis is on using an artificial ring and artificial chordae to repair the mitral valve physiologically, instead of using “resection and suture,” which has traditionally been the approach of skilled surgeons. In order to perform these surgical procedures with high accuracy, preoperative TEE measurements are essential.

Sizing the annuloplasty ring and determining the length of the artificial chordae are performed in diastole during hyperkalemic cardiopulmonary arrest. However, there was found to be a difference of a few millimeters between the systolic and diastolic cardiac cycles [9–11]. In addition, our previous research suggested that sizing of the mitral annuloplasty ring should be performed in the systole [12].

Therefore, it may be ideal if the ring size and artificial chordae length are determined based on measurements obtained using three-dimensional (3D) and two-dimensional (2D) TEE while the heart is still beating.

In this study, our group used TEE during R-MVP for Barlow’s disease to predict the optimal artificial ring size and artificial chordae length and thereby prevent residual mitral regurgitation (MR). Postoperatively, we evaluated the residual regurgitation of the mitral valve and examined whether the anesthesiologist’s predict repair plan was consistent with the surgeon’s surgical plan.

## Methods

This study was approved by the Institutional Review Board of our institute (approval no. 19-193). We recruited 10 consecutive patients diagnosed with severe MR due to Barlow’s disease who had undergone R-MVP at our hospital between June 2018 and April 2019.

**Fig. 1:**
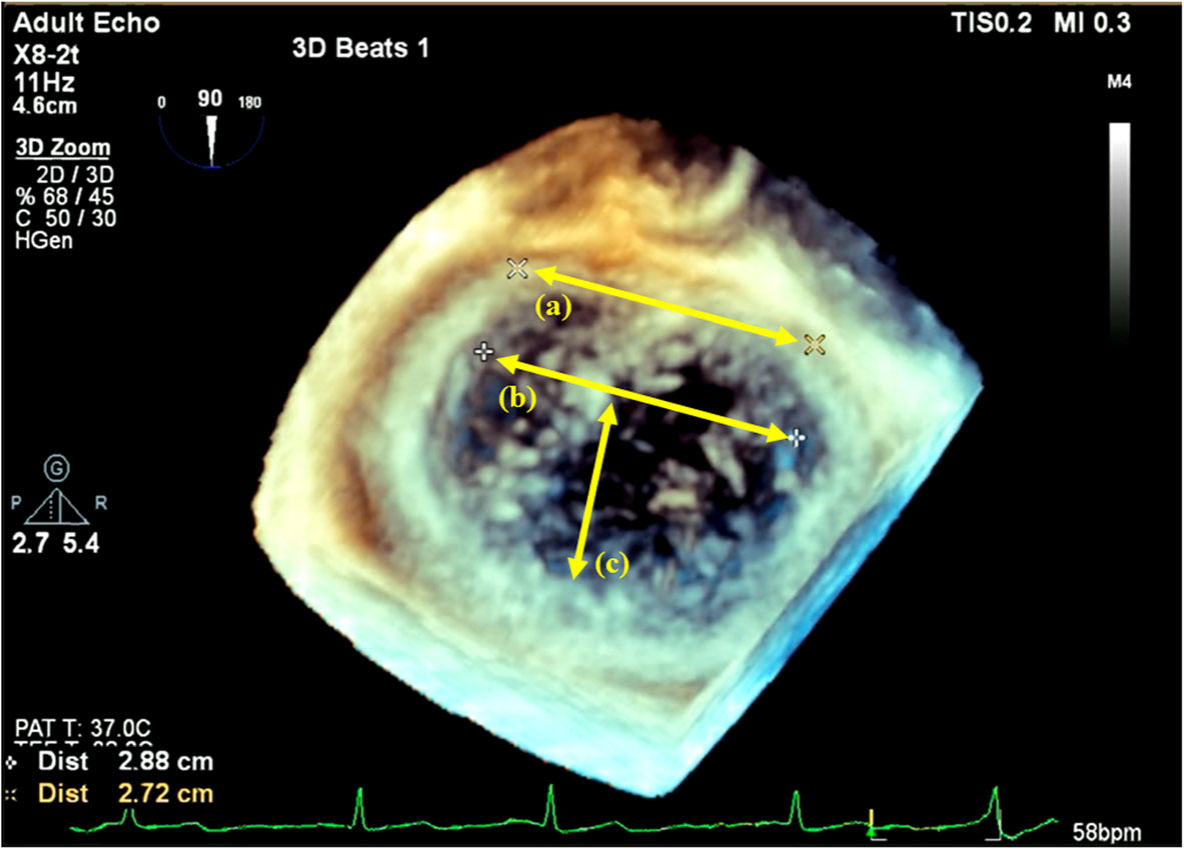
Sizing of the mitral valve when treating Barlow’s disease was performed using three-dimensional TEE at the end of systole. The optimal ring size was determined mainly with reference to the intercommissural (IC) distance. When changing the predetermined ring size, the height of the A2 segment of the anterior leaflet (A-height) was considered. In cases with a short A-height and long IC, size was determined by considering the intertrigonal (IT) distance, longitudinal diameter (LD), and transverse diameter (TD). Abbreviations: TEE, transesophageal echocardiography

**Fig. 2:**
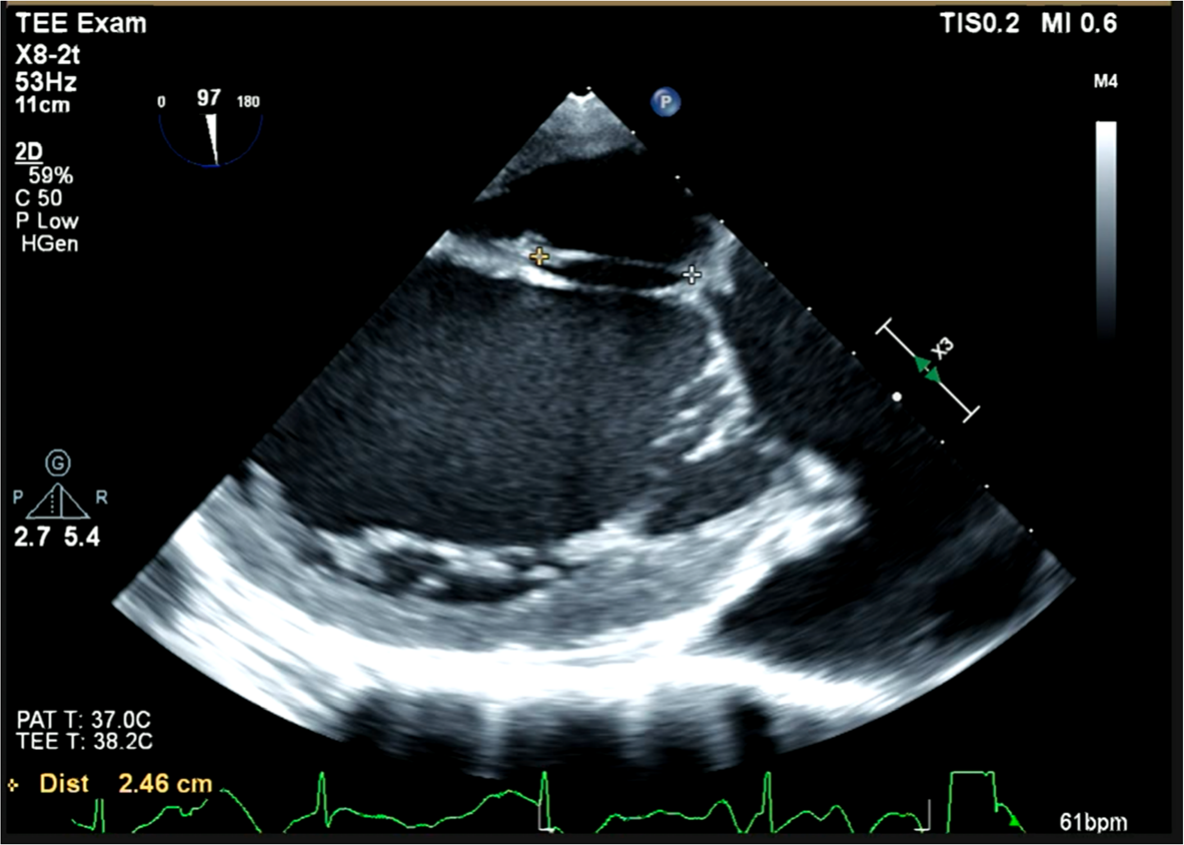
Two-dimensional, transgastric, two-chamber view to determine the optimal artificial chordae length. The optimal length of the artificial chordae was defined as equivalent to the length of the native secondary chordae adjacent to the prolapse site

After general anesthesia induction and once the patients’ hemodynamics were stable, a single anesthesiologist certified by the Japanese Board of Perioperative Transesophageal Echocardiography measured the size of the mitral valve at the end of systole using 3D- and 2D-TEE, EPIQ CVxTM with the x7-2t and x8-2t matrix-array transducers (Phillips, Amsterdam, Netherlands), as shown in Figs. 1 and 2. Based on the 3D and 2D TEE measurements, the anesthesiologist predicted the optimal annuloplasty ring size as well as the artificial chordae length and number for repair of the mitral valve, as follows. These measurement results were not disclosed to the surgeon.

### The optimal annuloplasty ring size

Using a 3D-zoom mode, at the end of systole, the mitral valve was visualized in enface view and the following parameters were measured directly: intercommissural (IC) distance, height of the A2 segment of the anterior leaflet (A-height), intertrigonal (IT) distance, longitudinal diameter (LD), and transverse diameter (TD) (Fig. 1). To avoid parallax errors, before constructing the 3D image, we matched the ultrasound beam direction perpendicular to the annulus with two left ventricular zoom images obtained in the orthogonal plane, as in a previous study [12]. The IC distance measured by 3D-TEE was used to predict the size of Physio II^TM^, Cosgrove^TM^, and CG Future^TM^ rings because the size of their annuloplasty ring sizers is based on the IC distance. By contrast, the size of the Taylor ring sizer is based on the IT distance. If the A-height was greater than two-thirds of the IC distance, the suggested ring size was increased by one.

### The length and numbers of artificial chordae

The optimal length of artificial chordae was defined as equivalent to the length of the native secondary chordae adjacent to the prolapse site at the end of systole; this length was decided by the anesthesiologist using a 2D, transgastric, two-chamber view (Fig. 2). The number of loop sets used in the repair plan and the length of the artificial chordae were commanded from the anesthesiologist to the assistant surgeon. The loop set was prepared before the start of surgery by an assistant surgeon using Gore-Tex (WL Gore and Associates, Flagstaff, AZ, USA).

Postoperatively, we evaluated whether the size of the implanted annuloplasty ring and the length and number of artificial chordae were matched to those planned by the anesthesiologist. The anesthesiologist’s repair plan was deemed match when all three of the following conditions were satisfied: (1) the anesthesiologist and surgeon selected an artificial ring of the same size, (2) there was no change the number of pre-planned loop sets and the length of the artificial chordae intraoperatively, (3) at the end of cardiopulmonary bypass (CBP), the residual MR was less than mild [13].

After the R-MVP was performed according to the preoperative plan, and after CBP weaning, and attainment of stable hemodynamics, the anesthesiologist evaluated the degree of MR as none, mild, mild to moderate, mild to moderate, or severe based on the guidelines of the American Society of Echocardiography [13].

## Results

During this period, 10 patients underwent R-MVP. All data are shown as the median (interquartile range) below. Operating time was 195 (181-239) min, CPB time was 136 (89-183) min, and aortic clamp time was 88 (42-134) min. One patient needed re-CPB. Systolic anterior movement (SAM) of the mitral valve was observed immediately after plastic surgery in two patients but disappeared in both cases with administration of phenylephrine and reduction of the dopamine dose. In seven of 10 cases, a mitral valve repair plan was fully matched between the anesthesiologists and surgeons. Postoperative MR was not observed in six of seven patients. All patients were extubated on the day of surgery or early the next day. The average number of prolapse sites per patient was 3.0 (2.3–3.0), and all patients were diagnosed with Barlow’s disease by direct intraoperative findings.

Details of the mitral valve measurements for individual patients and the planned ring sizes by the anesthesiologist are shown in Table 1. The mean number of loop sets used was 6.0 (4.5–8.0), and the mean length of artificial chordae was 20.0 mm (17.5–20.0). Two patients required changes in the length and number of artificial chordae prepared by the assistant surgeon: the addition of an artificial chord in one patient, and the extension of a chord in one patient. The annuloplasty ring sizes used were 28 mm in six patients, 30 mm in five, 32 mm in three, 33 mm in three, 34 mm in three, 35 mm in two, and 36 mm in one. The types of artificial rings used were PHYSIO (Edwards, USA) in four patients, Tailor (Medtronic, Ireland) in three patients, and CG Future (Medtronic) in three patients. The ring size planned by the anesthesiologist and the size implanted by surgeons matched in 80% (8/10) of patients (Table 2). Postoperative transthoracic echocardiography showed no regurgitant MR in nine patients, and mild residual MR in the other patient.

**Table 1:** Detailed mitral measurements and the planned ring sizes by the anesthesiologist

Cases	Intercommissural distance	A-height	Intertrigonal distance	Longitudinal diameter	Transverse diameter	Planned ring size
1	32.7	23.1	32.1	33.8	40.9	34
2	34.2	25.0	33.9	31.3	37.5	34
3	32.3	20.8	31.9	31.5	40.0	32
4	35.0	22.7	34.0	36.4	42.0	35
5	34.5	19.0	34.7	34.5	50.7	35
6	30.4	20.2	29.0	32.3	40.4	30
7	31.8	23.0	31.0	32.8	42.9	32
8	34.3	22.9	31.9	35.5	41.5	35
9	32.3	20.9	31.1	34.8	37.0	32
10	35.6	22.0	34.9	36.7	38.2	36
Median (IQR)	33.5 (32.3–34.5)	22.4 (20.8–23.0)	32.0 (31.3–34.0)	34.2 (31.3–32.4)	40.7 (38.7–41.9)	

Each case: mm

Abbreviations: *A-height* the height of A2 segment of anterior leaflet

**Table 2:** Departure site of the mitral valve, loop set count, planned and actually used ring sizes, changes in surgical plan, and residual postoperative MR

Cases	Prolapse site	Suggested loop (set)	Suggested AC length (Pm/Al)	Ring size suggested/implanted	Difference between suggested and implanted	Residual MR
1	P1P2	4	13/17	34/34	-	-
2	A1P1	4	17/19	34/34	-	-
3	A3P3Pcom	6	16/18	32/32	3-mm extension of artificial chordae	-
4	P1P2P3	8	20/21	35/35	-	-
5	A1A2	8	21/24	35/35	-	Mild
6	A1A2A3	6	20/22	30/30	-	-
7	A2A3P2P3	6	20/21	32/30	Changed ring size	-
8	A2A3P2P3	4	21/-	35/33	Changed ring size, one additional 19-mm artificial chord	-
9	P1P2P3	8	20/20	32/32	-	-
10	P1P2P3	8	20/20	36/36	-	-

Each case: mm

Abbreviations: *A* anterior leaflet; *P* posterior leaflet; *Com* commissure; *AC* artificial chordae; *Pm* posteromedial papillary muscle; *Al* anterolateral papillary muscle; *MR* mitral regurgitation

## Discussion

In this study, the annuloplasty ring sizes and artificial chordae lengths predicted by the anesthesiologist matched those that were actually implanted in 70% (7/10) of patients, of whom 86% (6/7) had no residual MR (Table 2). In addition, postoperative regurgitation remained in only one of 10 patients.

TEE is known to be very useful for immediate intraoperative evaluation in robot-assisted surgery [14–16]. Three-dimensional TEE is highly effective for observing the mitral complex structure [17]. Since Barlow’s disease is characterized by multiple prolapse sites, the repair plan differs from that used for local prolapse, and various mitral valve repair techniques have been proposed [18–23]. No previous studies have used TEE to predict the annuloplasty ring size and artificial chordae length during robotic, beating heart surgery for Barlow’s disease. With the loop technique, the mitral valve may cease functioning if the artificial chordae length is just 10% longer [24].

The advantage of the loop technique is that it allows re-try with reduced manipulation of the papillary muscles [11, 25]. If possible, however, chordae of the correct length should be implanted with as few trials as possible. Since the myocardium is relaxed during hyperkalemic cardiac arrest, it is difficult to determine the chordae length that will be appropriate when the heart is beating; therefore, estimating the optimal length based on beating heart TEE measurements may be more useful than direct sizing under cardiac arrest [12]. In this study, predicting the optimal annuloplasty ring size and optimal artificial chordae length using 3D-TEE resulted in successful mitral valve repair in six of seven patients. This suggests that 3D-TEE measurements may provide useful information in R-MVP for Barlow’s disease.

During the estimation process, we recommend avoiding the use of excessively long artificial chordae to be connected to the posterior leaflet in R-MVP. This is because in Barlow’s disease, the posterior apex is large; thus, if the artificial chordae connected to the posterior apex are too long, the coaptation line is positioned relatively anteriorly, increasing the risk of MR due to SAM when CBP is terminated [26]. In this study, two of 10 patients developed SAM, an undesirable complication after mitral valve repair, immediately after cardiopulmonary weaning. Preoperative prediction of the optimal artificial chordae length by TEE should help to avoid SAM.

Several limitations should be acknowledged. First, this was an observational study rather than a randomized controlled trial, and the ring sizes and artificial chordae lengths that were predetermined by the anesthesiologist using TEE were not ultimately adopted in all patients. Second, the length of artificial chordae should be compared for the length measured by other methods such as computed tomography (CT), magnetic resonance imaging (MRI), or direct measurement at the surgical field. In our study, no such comparison was made; the validity of the results may be small. Third, TEE measurements were performed by a single expert, but different results may be obtained depending on the skill of the TEE operator. Inter-operator reproducibility is relatively low, and there is a difference in accuracy between experts and beginners [12, 18]. Finally, the number of target patients was small.

## Conclusion

In conclusion, during R-MVP for Barlow’s disease with complex, multi-site prolapse, TEE was useful for selecting the appropriate artificial chordae lengths and artificial ring sizes. Therefore, TEE measurements should be considered for developing a more accurate mitral valve repair plan.

## Supplementary information

**Additional file 1:.** Table S1. Data set

## Data Availability

Please see the attached file.

## Abbreviations

TEE: Transesophageal echocardiography
MICS: Minimally invasive cardiac surgery
MICS-MVP: Minimally invasive mitral valvuloplasty
R-MVP: Robot-assisted mitral valvuloplasty
2D: Two-dimensional
3D: Three-dimensional
MR: Mitral regurgitation
IC: Intercommissural
A-height: A2 segment of the anterior leaflet
IT: Intertrigonal
LD: Longitudinal diameter
TD: Transverse diameter
CBP: Cardiopulmonary bypass
SAM: Systolic anterior movement
CT: Computed tomography
MRI: Magnetic resonance imaging
