# Supplementary material for: Transesophageal echocardiography in robot-assisted mitral valve repair for Barlow’s disease: usefulness for predicting artificial ring size and artificial chordae length using the loop technique
Source: JA Clin Rep. 2020 Jul 25;6:56. doi: 10.1186/s40981-020-00363-2 (PMC7382665; doi:10.1186/s40981-020-00363-2)
Supplement: Supplementary file 1 — Additional file 1:. Table S1. Data set [file 40981_2020_363_MOESM1_ESM.docx]

Criteria of causal inference

| Ní Bhrolcháin and Dyson (2007) | Time order | Rothman and Greenland ([1998](#B57)) | Strength |
| --- | --- | --- | --- |
|  | Contiguity |  | Consistency |
|  | Duration |  | Specificity |
|  | Distinctiveness |  | Temporality |
|  | Direction |  | Biologic gradient |
|  | Proportionality |  | Plausibility |
|  | Recurrence |  | Coherence |
|  | No cause, no effect |  | Experimental evidence |
|  | Mechanism |  | Analogy |
|  | No alternative |  |  |
